# Mathematically universal and biologically consistent astrocytoma genotype encodes for transformation and predicts survival phenotype

**DOI:** 10.1063/1.5037882

**Published:** 2018-09-19

**Authors:** Katherine A. Aiello, Sri Priya Ponnapalli, Orly Alter

**Affiliations:** 1 Scientific Computing and Imaging Institute, University of Utah, Salt Lake City, Utah 84112, USA; 2 Department of Bioengineering, University of Utah, Salt Lake City, Utah 84112, USA; 3 Huntsman Cancer Institute and Department of Human Genetics, University of Utah, Salt Lake City, Utah 84112, USA

## Abstract

DNA alterations have been observed in astrocytoma for decades. A copy-number genotype predictive of a survival phenotype was only discovered by using the generalized singular value decomposition (GSVD) formulated as a comparative spectral decomposition. Here, we use the GSVD to compare whole-genome sequencing (WGS) profiles of patient-matched astrocytoma and normal DNA. First, the GSVD uncovers a genome-wide pattern of copy-number alterations, which is bounded by patterns recently uncovered by the GSVDs of microarray-profiled patient-matched glioblastoma (GBM) and, separately, lower-grade astrocytoma and normal genomes. Like the microarray patterns, the WGS pattern is correlated with an approximately one-year median survival time. By filling in gaps in the microarray patterns, the WGS pattern reveals that this biologically consistent genotype encodes for transformation via the Notch together with the Ras and Shh pathways. Second, like the GSVDs of the microarray profiles, the GSVD of the WGS profiles separates the tumor-exclusive pattern from normal copy-number variations and experimental inconsistencies. These include the WGS technology-specific effects of guanine-cytosine content variations across the genomes that are correlated with experimental batches. Third, by identifying the biologically consistent phenotype among the WGS-profiled tumors, the GBM pattern proves to be a technology-independent predictor of survival and response to chemotherapy and radiation, statistically better than the patient's age and tumor's grade, the best other indicators, and *MGMT* promoter methylation and *IDH1* mutation. We conclude that by using the complex structure of the data, comparative spectral decompositions underlie a mathematically universal description of the genotype-phenotype relations in cancer that other methods miss.

## INTRODUCTION

Recurring DNA alterations have been recognized as a hallmark of cancer for over a century^1^ and observed in astrocytoma brain cancer for decades, without being translated into clinical use.^2^ Meanwhile, the prognosis, diagnosis, and treatment of astrocytoma have remained largely unchanged. Temozolomide, the one drug that progressed from trials to standard of care, modestly improves the one-year median survival time of grade IV astrocytoma, i.e., glioblastoma (GBM), by less than three months.^3^ This is despite advances in genomic profiling technologies and the growing number of publicly available genomic data.^4,5^ Only recently, a copy-number genotype predictive of an astrocytoma survival phenotype was discovered and only by using the generalized singular value decomposition (GSVD) to compare patient-matched primary adult GBM and, separately, grades III and II, i.e., lower-grade astrocytoma (LGA) tumor and normal genomes, profiled by Agilent comparative genomic hybridization (CGH) and Affymetrix single nucleotide polymorphism (SNP) microarray platforms, respectively.^6,7^ Note that primary GBM and LGA are different types of cancers. Their histopathologies overlap, and GBM is distinguished from LGA by the presence of necrosis or microvascular proliferation in the tumor. Their epidemiologies, however, differ, including the distributions of the results of existing tests, i.e., for *MGMT* promoter methylation and *IDH1* mutation, and, therefore, also the distributions of treatments, i.e., chemotherapy and radiation.^8^

To test the mathematical universality and biological consistency of the tumor-exclusive genotype and phenotype, here we use the GSVD to additionally compare whole-genome sequencing (WGS) read-count profiles of astrocytoma tumor and patient-matched normal DNA^9^ from the Cancer Genome Atlas (TCGA). We used the same computational workflow to construct the WGS astrocytoma set of patients as we previously used to construct the Agilent GBM and Affymetrix LGA discovery and validation sets (Methods and Fig. S1 in the supplementary material). The resulting tumor and normal datasets have the structure of two matrices of *N *=* *85 matched columns, i.e., patients, and *M*_1_ = 2 827 037 and *M*_2_ = 2 828 152 rows, i.e., tumor and normal 1K-nucleotide bins^10,11^ (Dataset S1).

The WGS technology complements the CGH and SNP microarray platforms to represent the main genomic profiling technologies. Note that each technology relies on a specific experimental design and a specialized computational protocol, which is sensitive to perturbations to the data, e.g., due to changes in the experimental batch or the computational preprocessing.^12–14^ This has contributed to a low reproducibility, <70% between technical replicates of the same sample and <50% between computational assessments of the same raw data, in assigning copy-number variations (CNVs) in normal DNA^15^ or copy-number alterations (CNAs) in tumor DNA. The WGS set of bins, while different from the Agilent CGH and Affymetrix SNP sets of probes, provides a high-resolution representation of the human genome, like the CGH and SNP sets. The ≈2.8M bins, across the autosome and the X chromosome, include almost all of the 213K CGH and 934K SNP probes. In addition, the bins fill in gaps in the genome which are not covered by either set of probes, mostly in genomic regions of constitutive heterochromatin domains, e.g., the centromeres and telomeres.

The WGS astrocytoma set of patients, while different from the mutually exclusive Agilent GBM and Affymetrix LGA discovery and validation sets of patients, statistically represents the astrocytoma patient population at large, like the GBM and LGA sets representing the GBM and LGA populations, respectively. The representation is in terms of both disease and normal phenotypes, e.g., gender and ethnicity, while reflecting biases against surgical resections in patients >75 years old or of diffuse tumors, which affect mostly GBM or LGA patients, respectively. The 85 WGS astrocytoma patients include ≈61%, 28%, and 11% primary GBM and grade III and II astrocytoma patients, diagnosed at the median ages of 60, 50, and 31 years, and with median survival times of 15, 58, and 63 months, respectively. *IDH1* mutation was detected in 15%, 48%, and 86% of the tested GBM and grade III and II astrocytoma patients, respectively. Treatment by chemotherapy was noted for 77% GBM and 55% LGA patients. There are 62% male and 38% female patients. Of the 85 WGS astrocytoma patients, 24, i.e., ≈28%, complement the discovery sets of 251 GBM and 59 LGA patients. Of these 24 patients, 14 complement the validation sets of 184 GBM and 74 LGA patients and include GBM and grade III and II astrocytoma patients.

The WGS astrocytoma tumor and patient-matched normal datasets, while different from the Agilent GBM and Affymetrix LGA datasets, represent a range of approaches to tissue collection from 1993 to 2012 and DNA extraction and genomic characterization, like the Agilent GBM and Affymetrix LGA datasets. Participating in generating the data were 18 TCGA tissue source sites (TSSs), two biospecimen core resources (BCRs), and three genomic characterization centers (GCCs), employing two different types of DNA sequencing instruments. Even while controlling for intratumor heterogeneity, TCGA parameters, e.g., the tumor sample's volume, can span approximately two orders of magnitude.

We find that first the GSVD identifies the same genotype-phenotype relation as significant in, and exclusive to, the WGS astrocytoma tumor relative to the patient-matched normal profiles, here as in the previous GSVDs of Agilent GBM and, separately, Affymetrix LGA tumor and normal profiles. The identification is invariably blind to, i.e., without *a priori* information about, the clinical labels of the patients, the experimental labels of the samples, or the genomic coordinates of the bins or probes. This identified relation is invariably robust to perturbations to the minimally preprocessed data and independent of intratumor heterogeneity as it is reflected in the TCGA parameters.

Second, independent of the profiling technology, the GSVD blindly separates the tumor-exclusive genotype-phenotype relation from experimental batch effects. Affecting the WGS data, here we find guanine-cytosine (GC) content variations across the genomes that vary in magnitude between TCGA GCC and TSS batches. Affecting the microarray data, previously we found batches of, e.g., hybridization dates, scanners, and plates. Additional separation is from normal relations that are conserved in the tumor, e.g., the X chromosome genotype and the gender phenotype. Note that depending on the technology, this relation is represented in the data as a male-specific deletion or a female-specific amplification of the X chromosome relative to the autosome or the normal male genome, respectively.

Third, the tumor-exclusive genotype invariably predicts the phenotype of astrocytoma survival and response to chemotherapy and radiation statistically better than and independent of any other indicator, test, and treatment, here, for the WGS astrocytoma set of patients, as it did previously for the mutually exclusive Agilent GBM and Affymetrix LGA discovery and validation sets of patients.

We, therefore, conclude that the tumor-exclusive genotype-phenotype relation is appropriate for the adult astrocytoma population at large and suitable for all genomic profiling technologies. That is, that the GSVD formulated as a comparative spectral decomposition underlies a mathematically universal description of the genotype-phenotype relations in astrocytoma.

## THE GSVD AS A COMPARATIVE SPECTRAL DECOMPOSITION

Given two column-matched but row-independent real matrices $D_{i}\in {\mathbb{R}}^{M_{i}\times N}$, each with full column rank $N\leq M_{i}$, the GSVD is an exact simultaneous factorization^16–19^

$$D_{i}=U_{i}\Sigma_{i}V^{T}=\sum_{n=1}^{N}\sigma_{i,n}u_{i,n}⊗v_{n}^{T},\;i=1,2,$$ (1)

where $U_{i}\in {\mathbb{R}}^{M_{i}\times N}$ are real and column-wise orthonormal and $V^{T}\in {\mathbb{R}}^{N\times N}$ is real, invertible, and with normalized rows. The 2 *N* positive generalized singular values are arranged in $\Sigma_{i}=\text{diag}(\sigma_{i,n})\in {\mathbb{R}}^{N\times N}$ in a decreasing order of the ratio $\sigma_{1,n}/\sigma_{2,n}$. The GSVD is unique up to phase factors of ±1 of each triplet of the corresponding column and row basis vectors, i.e., $u_{i,n}$ and *v_n_*, except in degenerate subspaces defined by subsets of pairs of generalized singular values of equal ratios, i.e., $\sigma_{1,n}/\sigma_{2,n}$. The GSVD generalizes the SVD from one to two matrices. Like the SVD, the GSVD is a mathematical building block of algorithms, e.g., for solving the problem of constrained least squares in algebra,^20^ and theories, e.g., for describing oscillations near equilibrium in classical mechanics.^21^

**FIG. 1.**
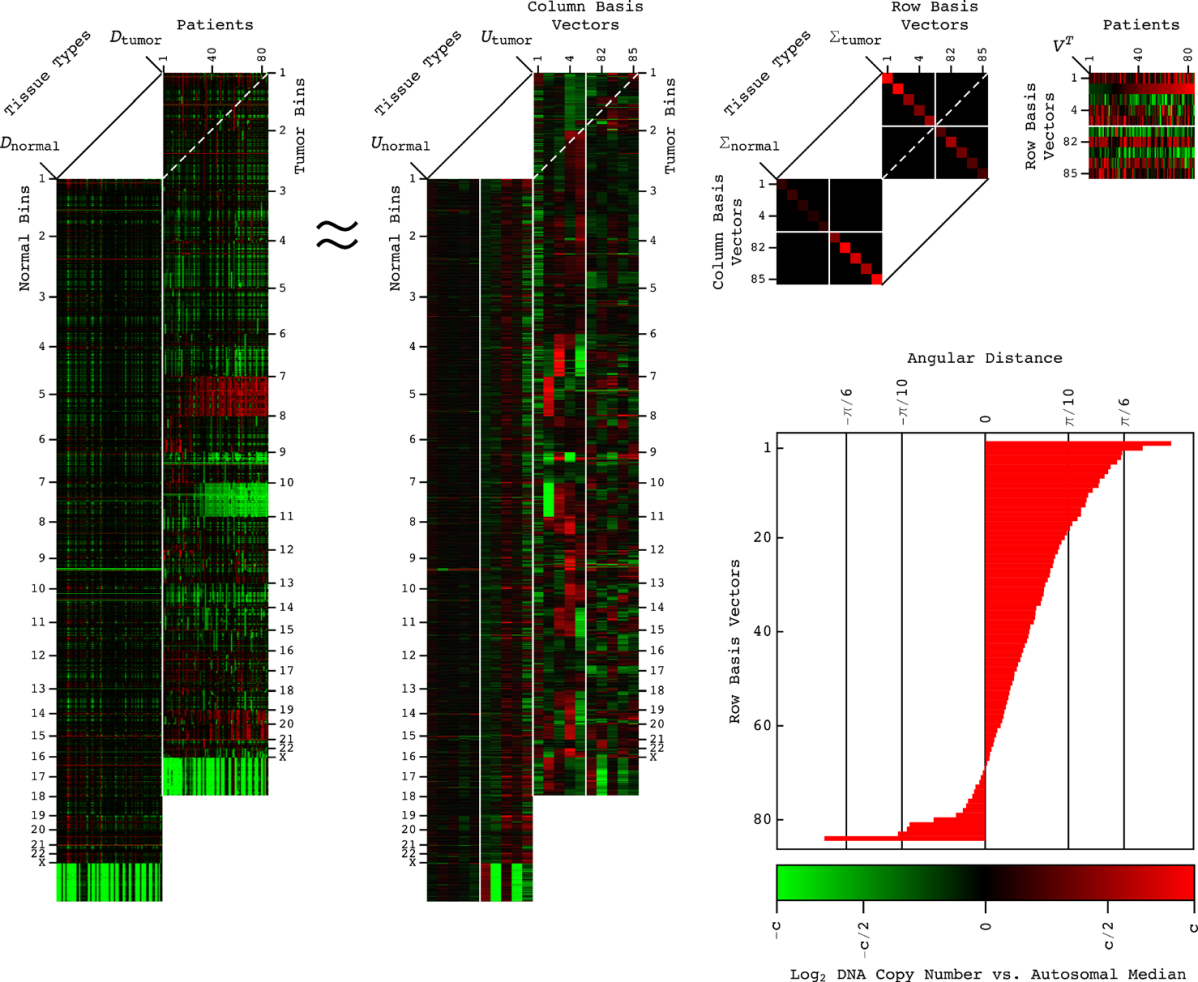
The GSVD of the WGS read-count profiles of patient-matched astrocytoma tumor and normal DNA. The GSVD is depicted in a raster display with the relative WGS read-count, i.e., DNA copy-number amplification (red), no change (black), and deletion (green). This GSVD depiction is denoted as approximate, even though the GSVD of Eq. (1) is exact, because only the first through the 5th and the 81st through the 85th row and the corresponding tumor and normal column basis vectors and generalized singular values are explicitly shown. The angular distances of Eq. (2) are depicted in a bar chart. The red and green contrasts for the datasets *D_i_*, the dataset-specific column basis vectors *U_i_* and generalized singular values Σ_*i*_, and the dataset-shared row basis vectors *V^T^*, are *c *=* *1, 750 and 0.0005, and 5, respectively.

We formulated the GSVD as a comparative spectral decomposition that can simultaneously identify the similarity and dissimilarity between two column-matched but row-independent matrices and, therefore, create a single coherent model from two datasets recording different aspects of interrelated phenomena.^22,23^ This formulation^24–27^ is possible because the GSVD is exact, exists, and has uniqueness properties that directly generalize those of the SVD^28,29^ (Theorem S1). The only assumption is that there exists a one-to-one mapping between the columns of the matrices but not necessarily between their rows. We defined the significance of the row basis vector *v_n_* and the corresponding column basis vector $u_{i,n}$ in the corresponding matrix *D_i_*, i.e., the “generalized fraction” $p_{i,n}$, to be proportional to the corresponding generalized singular value $\sigma_{i,n}$ and the “generalized normalized Shannon entropy” of *D_i_* to be proportional to the arithmetic mean of $p_{i,n}\;\text{log}\;p_{i,n}$ (Fig. S2). We defined the significance of *v_n_* and $u_{1,n}$ in *D*_1_ relative to that of *v_n_* and $u_{2,n}$ in *D*_2_, i.e., the “GSVD angular distance,” to be a function of the ratio $\sigma_{1,n}/\sigma_{2,n}$ that, from the cosine-sine decomposition, is related to an angle (Fig. 1)

$$-\pi /4<\theta_{n}=\arctan(\sigma_{1,n}/\sigma_{2,n})-\pi /4<\pi /4.$$ (2)

Note that the angular distances *θ_n_* are different from the principal angles corresponding to canonical correlations, as the GSVD is different from canonical correlations analysis (CCA).^30^

A unique row basis vector *v_n_* that is significant in either *D*_1_ or *D*_2_ and with an angular distance of $\theta_{n}\approx \pm \pi /4$, which corresponds to a ratio of $\sigma_{1,n}/\sigma_{2,n}\gg 1$ or ≪1, respectively, is mathematically approximately exclusive to either *D*_1_ or *D*_2_ and for consistency should be interpreted with the corresponding column basis vector $u_{1,n}$ or $u_{2,n}$ to represent phenomena exclusive to either the first or the second dataset. A unique row basis vector *v_n_* that is significant in both *D*_1_ and *D*_2_ and with an angular distance of $\theta_{n}\approx 0$, which corresponds to $\sigma_{1,n}/\sigma_{2,n}\approx 1$, is mathematically common to *D*_1_ and *D*_2_ and should be interpreted with both $u_{1,n}$ and $u_{2,n}$ to represent phenomena common to both datasets.

Mathematically invariant under the exchange of the two matrices or the reordering of the pairs of matched columns or the rows, the GSVD is also blind to the labels of the matrices, the columns, and the rows. These labels are only used to interpret the row and column basis vectors in terms of the phenomena recorded in the datasets.

## ASTROCYTOMA TUMOR-EXCLUSIVE GENOTYPE AND PHENOTYPE

The second most tumor-exclusive row basis vectors uncovered by the previous GSVDs of patient-matched Agilent GBM and, separately, Affymetrix LGA tumor and normal profiles are also the first and third most significant in the GBM and LGA tumor genomes, respectively. By using the clinical labels of the previous discovery sets of patients in survival analyses, these second row basis vectors were shown to separate subsets of patients of an approximately one-year median survival time from the complement subsets of median survival times of three years in GBM and five years in LGA. The corresponding second GBM and LGA tumor column basis vectors, i.e., patterns, were shown to similarly separate subsets of patients of an approximately one-year median survival time from the previous validation sets of patients.

By using the genomic coordinates of the microarray probes in segmentation analyses, the GBM and LGA patterns were shown to describe similar genome-wide patterns of co-occurring DNA CNAs that encode for opportunities for transformation via the Ras and Shh pathways. The GBM pattern, which encompasses the LGA pattern, such that these opportunities are enhanced in GBM relative to LGA, includes most CNAs that were known and several that were unrecognized in GBM prior to its discovery. We found that the GBM pattern predicts GBM survival statistically better than any one CNA that it identifies and that none of the previously known CNAs was correlated with GBM survival. We, therefore, suggested that the astrocytoma survival phenotype is an outcome of its global genotype.

**FIG. 2.**
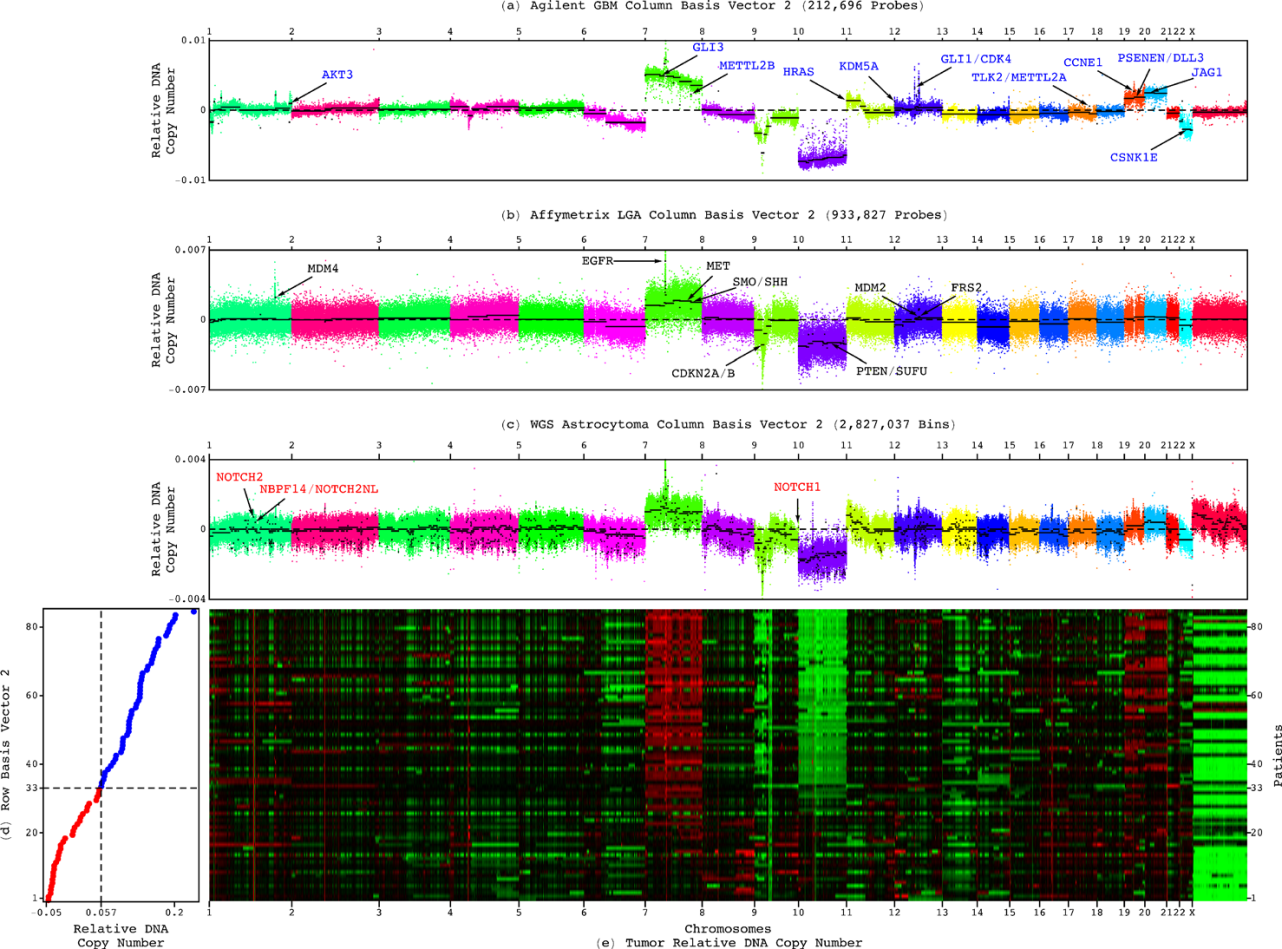
Astrocytoma tumor-exclusive genotype and phenotype. The similar genome-wide patterns of CNAs described by the second (a) Agilent GBM, (b) Affymetrix LGA, and (c) WGS astrocytoma tumor column basis vectors are depicted in plots of relative copy numbers, ordered and colored based upon genomic coordinates, and segmented by CBS (black lines), including GBM-specific (blue), GBM- and LGA-shared (black), or WGS technology-filled in (red) CNAs. (*d*) The second WGS astrocytoma row basis vector is depicted in a plot showing the classification of the 85 patients into low (red) or high (blue) superposition coefficients. (e) The WGS astrocytoma tumor dataset is depicted in a raster showing the tumor-exclusive genotype-phenotype relation.

Here, we find that the second tumor column basis vector uncovered by the GSVD of the WGS profiles is the second most significant in and exclusive to the astrocytoma tumor relative to the normal genomes and describes the same genotype (Fig. 2). To compare the corresponding WGS astrocytoma pattern to the Agilent GBM and Affymetrix LGA patterns, we used the genomic coordinates of the WGS bins and classified the 111 genomic segments of at least five Agilent probes in length, previously identified in the Agilent GBM pattern, as amplified, unaltered, or deleted in the WGS astrocytoma pattern in addition to the Affymetrix LGA pattern (Dataset S2). The classification is based upon the differences, in standard deviations, between the relative copy-number means of the segments and the autosome or the chromosomes. We find that the WGS astrocytoma pattern is approximately bounded above by the Agilent GBM and below by the Affymetrix LGA pattern; ≳83% of the segments that are amplified or deleted in the WGS astrocytoma pattern are a subset and a superset of, and of a lesser or greater magnitude than, those that are amplified or deleted in the Agilent GBM and Affymetrix LGA patterns, respectively.

### An approximately one-year median survival time phenotype

By using the clinical labels of the patients, we find that the WGS astrocytoma pattern is correlated with the same survival phenotype as the Agilent GBM and Affymetrix LGA patterns (Fig. S3). Of the 85 patients, 52 are classified as having high weights of the astrocytoma pattern in their tumor profiles based upon the superposition coefficients of the second tumor column basis vector in the column vectors of the tumor dataset. The vector that lists these coefficients is linearly proportional to the second row basis vector. Of the same 85 patients, 54, including 51, i.e., ≈98% of the 52, have high Pearson correlations of their tumor profiles with the pattern. We use the correlation cutoff of 0.15 and compute the coefficient cutoff by scaling 0.15 by the Frobenius norm of the vector that lists the correlations, as was previously established for the Agilent GBM discovery set of patients and validated for the Agilent GBM validation and Affymetrix LGA discovery and validation sets of patients.

In Kaplan-Meier (KM) survival analyses, the subsets of patients with high superposition coefficients and, separately, Pearson correlations are of an approximately one-year median survival time, statistically significantly shorter than the median survival time of five years of the complement subsets of patients. In Cox proportional hazards models, a high coefficient or, separately, correlation confers ≈8 times the hazard of a low coefficient or correlation, respectively.

### A genotype encoding for transformation via the Notch together with the Ras and Shh pathways

**FIG. 3.**
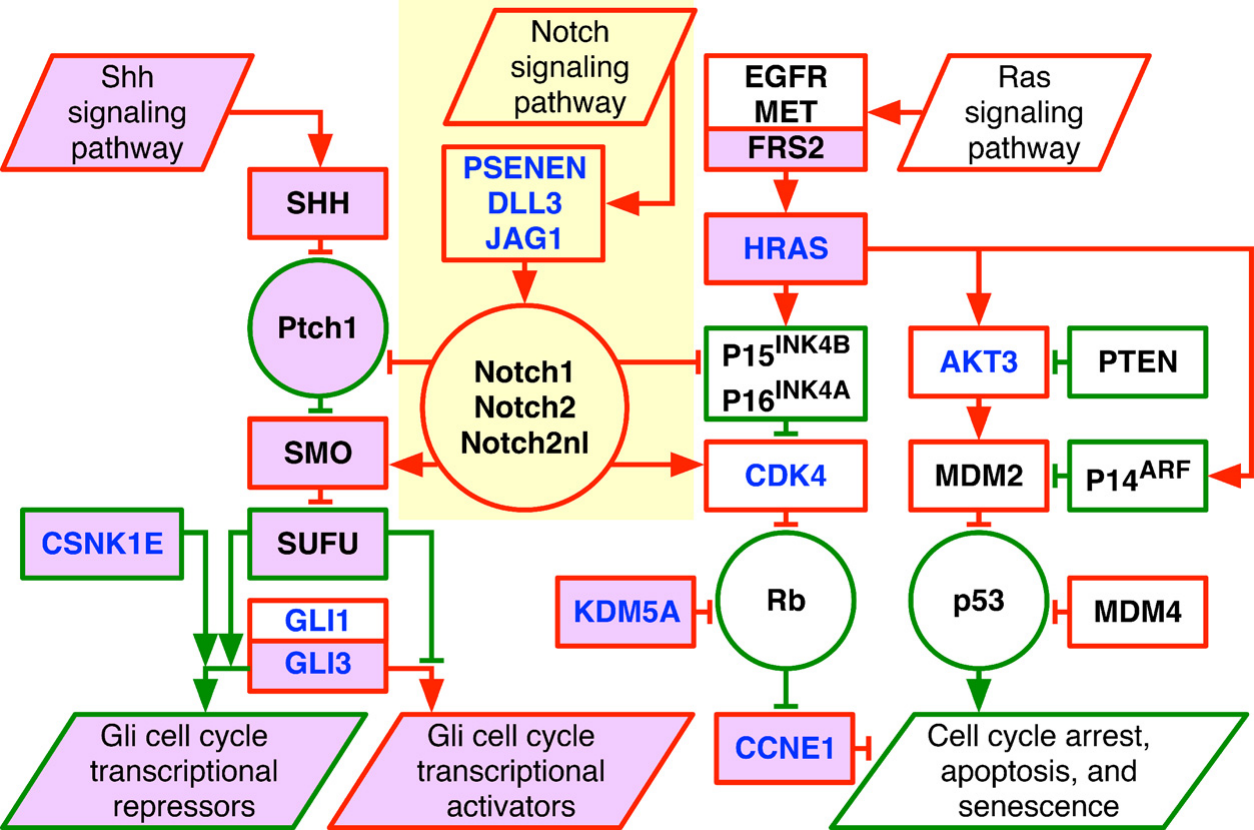
The astrocytoma tumor-exclusive genotype encodes for increased cell communication via the canonical Notch pathway in support of transformation via the Ras, Shh, and hominin-specific Notch pathways. The astrocytoma genotype is depicted in a diagram of the WGS technology-filled in Notch pathway (yellow) in addition to the microarray-described Ras and Shh pathways, which include CNAs unrecognized in GBM prior to the discovery of the GBM pattern (violet). Explicitly shown are amplifications (red) and deletions (green) of genes and transcript variants (rectangles), either GBM- and LGA-shared (black) or GBM-specific (blue), and relationships that directly or indirectly lead to increased (arrows) or decreased (bars) activities of the genes and transcripts, the tumor suppressor proteins p53, Rb, and Ptch1, and the oncoproteins Notch1, Notch2, and Notch2nl (circles).

By filling in gaps in the genome which are not covered by either the Agilent or the Affymetrix probes, the WGS astrocytoma pattern adds to the description of the genotype that corresponds to the one-year survival phenotype. We find amplifications previously unrecognized in astrocytoma which encode for increased cell communication via the canonical Notch pathway in support of transformation via the Ras and Shh and the hominin-specific Notch pathway (Fig. 3).

The largest of the 111 segments, which spans ≈79M nucleotides on chromosome 1 across the bands 1p31.1-q23.3, is classified as unaltered in the WGS pattern, the same as in the microarray patterns. The segment contains the two largest gaps between the microarray probes on chromosome 1. The largest, a 23M-nucleotide gap (1p11.2-q21), includes the centromere. Circular binary segmentation (CBS)^31^ of the WGS pattern identified a 21M-nucleotide segment (1p11.2-q12) within the gap, which is classified as amplified. At 739K nucleotides from the 5′ end of the gene *NOTCH2* (1p12-p11.2), the amplification is within its promoter region.^32^ Similarly, a 140K-nucleotide gap (9q34.3), which includes the 9q telomere, overlaps 79K of a 104K-nucleotide amplified segment in the promoter region of *NOTCH1* at 1.6M nucleotides from its 5′ end. These amplifications within the promoter regions, rather than of the genes, encode for overexpression of wild-type *NOTCH1/2*.^33,34^ Three genes in the core Notch pathway are on two of the 111 segments, which are approximately coextensive with 19q and 20p and are amplified in the GBM but not the LGA or astrocytoma patterns. The ligand-encoding *JAG1* and *DLL3* are involved in sending, and *PSENEN* in receiving, the Notch signals. These amplifications encode for overactivation of Notch in GBM. Note that the co-deletion of 1p and 19q, which can underactivate Notch, is associated with an oligodendroglioma brain cancer patient's longer survival.

Segmentation of the WGS pattern also identifies a 76K-nucleotide segment within the second largest gap on chromosome 1 (1q21.2). The segment, which is classified as amplified, maps to the neuroblastoma breakpoint family gene *NBPF14*, so-called because *NBPF1* (1p36.13) was discovered in a screen for genes disrupted by a translocation in a neuroblastoma brain cancer patient's normal genome.^35^ The segment includes 38 repeats of a 1.5K-nucleotide sequence that encodes for a copy of the protein domain of unknown function 1220 (DUF1220).^36^ At 2.3M nucleotides from the 5′ end of the hominin-specific *NOTCH2NL* (1q21.1), the amplification is within its promoter region and encodes for its overexpression.

Overactivation of the canonical Notch pathway supports human normal to tumor cell transformation via the Ras and Shh and the hominin-specific Notch pathway. In response to Ras-mediated growth signals, wild-type *NOTCH1/2* upregulate the cell cycle-promoting cyclin-dependent kinase (CDK) encoded by *CDK4* and blocks the cell cycle arrest, apoptosis, and senescence-promoting CDK inhibitors p16^INK4A^ and p15^INK4B^ encoded by *CDKN2A/B*.^37–40^ Note that in the absence of CDK inhibitors, DNA-damaged cells acquire deformed polyploid nuclei.^41,42^ In response to Shh-mediated developmental signals, *NOTCH1/2* facilitate the clearance of the tumor suppressor Ptch1, the concurrent accumulation of the Shh signal-transducing protein encoded by *SMO*, and the increased downstream conversion of the proteins encoded by the oncogenes *GLI1/3* into cell cycle transcriptional activators.^43,44^ Note that Notch is critical for an Shh-induced medulloblastoma brain cancer tumor's development.^45^

In the hominin-specific Notch pathway, *NOTCH2NL* can act as a ligand-independent *NOTCH1/2*.^46^ Note that overexpression of *NOTCH2NL* and gain of DUF1220 are associated with an increased brain size, both developmentally within the human and evolutionarily within the primate population.^47^

We also find consistency between the DNA CNAs and mRNA expression, which additionally supports the astrocytoma tumor-exclusive genotype-phenotype relation.^48^ Of the 29 genes highlighted, 19 are overexpressed or underexpressed in the subset of tumors that have high weights of the WGS astrocytoma pattern in their profiles, with the corresponding Mann-Whitney-Wilcoxon (MWW) *P*-values <0.05. This subset of tumors corresponds to the subset of patients that have the approximately one-year survival phenotype. Of these 19 genes, 16, i.e., ≈84%, consistently map to amplifications or deletions in the tumor-exclusive genotype (Figs. S4–S7).

## BLIND SEPARATION FROM NORMAL AND EXPERIMENTAL SOURCES OF THE COPY-NUMBER VARIATION

By using the experimental labels of the DNA samples, we find that the GSVD blindly, i.e., without *a priori* information, separates the astrocytoma tumor-exclusive genotype and phenotype from CNVs common to the normal and tumor genomes and from experimental variations specific to the minimally preprocessed WGS profiles. These include the effects of the GC content variations across the tumor and normal genomes that vary in magnitude between experimental batches. The first tumor and 85th normal column basis vectors are the most significant in and exclusive to and are correlated with the fractional GC content across the tumor and normal genomes, respectively, with both correlations ≳0.78 and both MWW *P*-values $<10^{-10^{5}}$ (Figs. S8–S10). Both vectors roughly describe frequent spikes of reduced copy numbers superimposed on an invariant baseline in agreement with the polymerase chain reaction (PCR) amplification-dependent WGS technology underestimating the abundance of GC-poor sequences. The corresponding first and 85th row basis vectors are correlated with experimental variations in the GCC of the tumor and TSS of the normal DNA with both hypergeometric and both MWW *P*-values <10^–2^ (Fig. S11).

The 82nd row basis vector is the second and fifth most significant in the normal and tumor genomes, respectively, and approximately common to both. The vector classifies the patients by gender with both hypergeometric and MWW *P*-values <10^–13^ (Fig. S12). Both normal and tumor 82nd column basis vectors describe a deletion of the X chromosome with both MWW *P*-values $<10^{-10^{4}}$ (Figs. S13–S15). While the deletion is dominant in the normal and tumor genomes of the 53 male patients, it is missing from the astrocytoma pattern, where the X chromosome is classified as unaltered, the same as in the GBM and LGA patterns.

## THE TUMOR-EXCLUSIVE GENOTYPE PREDICTS THE SURVIVAL PHENOTYPE STATISTICALLY BETTER THAN ANY OTHER INDICATOR

**FIG. 4.**
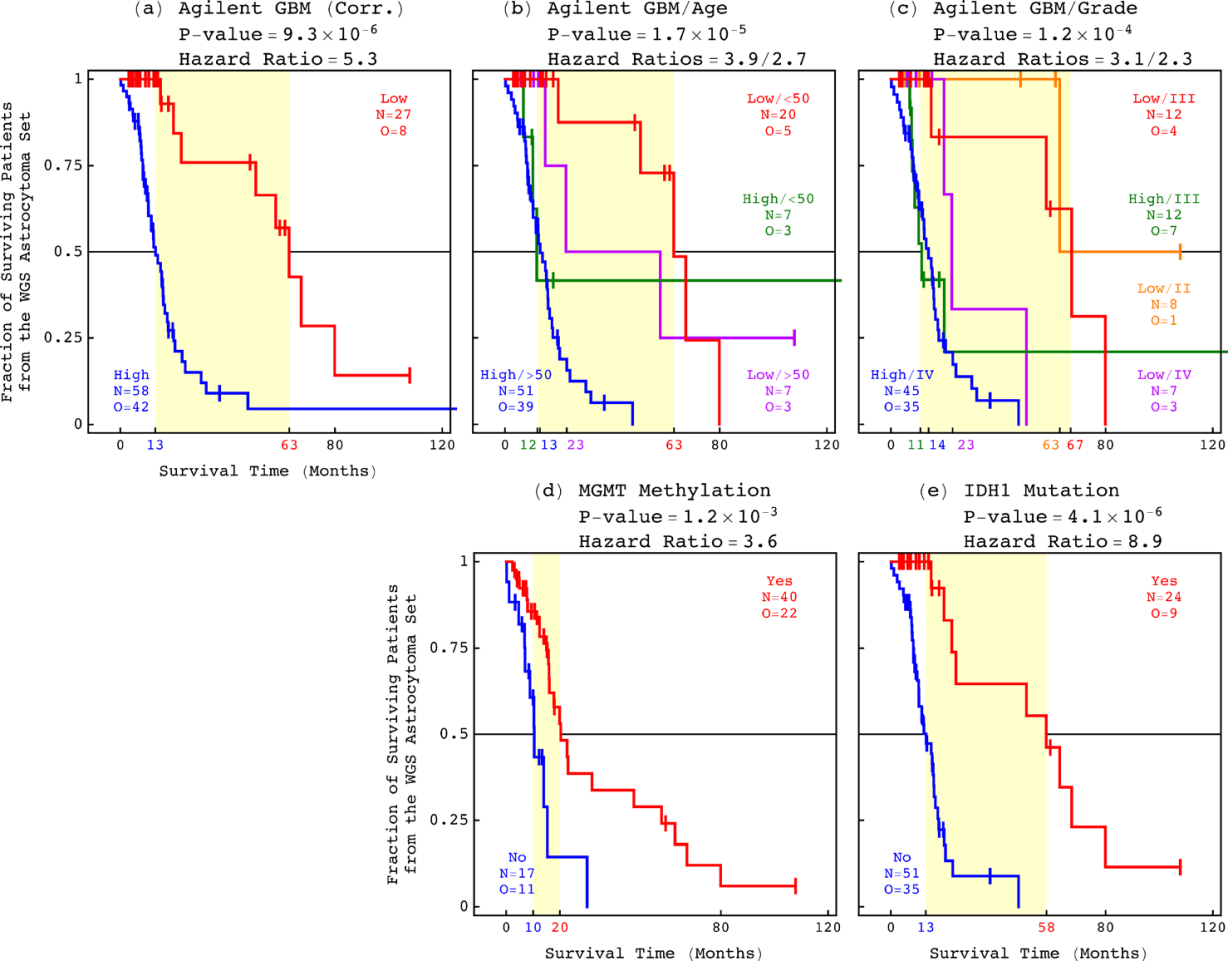
Survival analyses of the WGS astrocytoma patients. The classifications of the 85 patients based upon (a) the Agilent GBM pattern and, in addition, (b) age or (c) grade, or (d) MGMT promoter methylation or (e) *IDH1* mutation are depicted in KM curves highlighting median survival time differences (yellow) with the corresponding log-rank *P*-values and Cox hazard ratios.

Because the Agilent GBM pattern encompasses the WGS astrocytoma and Affymetrix LGA patterns in the number and magnitude of CNAs and because it was derived from the largest discovery set, i.e., of 251 patients, we additionally classified the 85 WGS astrocytoma patients based upon the correlations of the Agilent GBM pattern with their WGS astrocytoma tumor profiles. We find that the Agilent GBM pattern predicts survival statistically better than and independent of the best other indicators, i.e., the patient's age and tumor's grade^49^ and survival and response to treatments, i.e., chemotherapy and radiation, better than the existing tests, i.e., for MGMT promoter methylation and *IDH1* mutation.^50,51^ In KM analyses and Cox models of the patients, the pattern identifies the biologically consistent survival phenotype with greater median survival time differences, hazard ratios, and concordance indices, i.e., accuracies, and lesser log-rank *P*-values than either indicator or test (Fig. 4), and, in KM analyses and Cox models of the treated patients, better than either test (Fig. S16). The bivariate hazard ratios of the pattern and either indicator are within the 95% confidence intervals of the corresponding univariate ratios (Table S1). The pattern is also independent of intratumor heterogeneity as it is reflected in the TCGA parameters of the tumor sample's volume, the slide's percent tumor cells and percent tumor nuclei, the portion's weight, and the analyte's and aliquot's DNA concentrations.

This is consistent with the classifications based upon the WGS astrocytoma pattern, where the median survival time differences are the same and the hazard ratios are within the 95% confidence intervals of those based upon the Agilent GBM pattern. This is also consistent with the classifications of an Affymetrix set of 497 astrocytoma patients and, separately, an Agilent set of 364 GBM patients, from the previous discovery and validation sets of GBM and LGA patients, based upon their Affymetrix and Agilent tumor profiles, respectively, where the pattern is independent of each treatment, indicator, and test (Figs. S17 and S18, Tables S2 and S3, and Datasets S3 and S4).

That the tumor-exclusive genotype-phenotype relation is statistically independent of the current indicators, tests, and treatments of astrocytoma implies that the information contained in the relation is not currently being used in clinical practice. This information includes, e.g., biochemically putative drug targets and combinations of drug targets that are predicted to be correlated with outcome. By using this information in clinical practice, therefore, it can be expected to improve the prognostics, diagnostics, and therapeutics of the disease.

## DISCUSSION

That the astrocytoma tumor-exclusive genotype-phenotype relation is invariably uncovered by, and only by, the GSVD, independent of the profiling technology and the astrocytoma grade, highlights the role of mathematics in genomic data science and machine learning. Unlike most other analyses, the GSVD uses minimally preprocessed genomic data without feature engineering. This accounts for the robustness of the GSVD to perturbations to the data and is possible because of its scalability to petabyte-sized data. Other analyses often standardize the data based upon assumptions, which may confound the data and contribute to the low reproducibility noted in genomic profiling.

Unlike most other analyses, the GSVD uses the patient-matched normal data to analyze the tumor data, including tumor genomic regions of normal CNVs, e.g., the X chromosome. This makes the GSVD sensitive to robust genotype-phenotype relations in small discovery sets of only, e.g., 251, 59, and 85 patients, and possibly imbalanced validation sets of, e.g., 184 and 74 patients, with large genomic profiles of, e.g., 213K, 934K, and 2.8M probes or bins each. This is possible because the GSVD uses the structure of the tumor and normal datasets, of two column-matched but row-independent matrices, in the blind source separation (BSS)^52–64^ of the tumor-exclusive from the normal genotype-phenotype relations and from experimental batch effects. Patient-matched normal CNVs are often missing from other analyses of tumor CNAs, even though CNVs overlap ≈12% of the normal human genome,^65^ where they are 10^2^–10^4^ times more frequent than point mutations,^66^ and are associated with both tumor and normal development.^67–69^ When other analyses use patient-matched normal data, it is to standardize the tumor data. This reduces the structure of the data to that of one matrix, and some of the information regarding the similarity and dissimilarity between the tumor and normal genomes may be lost.

The GSVD as a comparative spectral decomposition^22,23^ has been extended from two to multiple matrices and, separately, two tensors.^24–26^ A recent tensor GSVD comparison of ovarian cystadenocarcinoma tumor and patient- and microarray platform-matched normal copy-number profiles uncovered chromosome arm-wide patterns of tumor-exclusive platform-consistent CNAs that predict survival and response to chemotherapy. We conclude that comparative spectral decompositions, such as the GSVD, underlie a mathematically universal description of the genotype-phenotype relations in cancer that other methods miss.

## METHODS

See supplementary material for the Methods section.

### Ethics approval

Ethics approval was not required to perform this research.

## SUPPLEMENTARY MATERIAL

See supplementary material for the Methods section, Figs. S1–S18, and Tables S1–S3, and Datasets S1–S4, also available at https://alterlab.org/astrocytoma_genotype-phenotype/.

## References

[1] T. Boveri , *Concerning the Origin of Malignant Tumours* ( Gustav Fischer Verlag, Jena, Germany, 1914)

[2] R. G. Weber , C. Sommer , F. K. Albert , M. Kiessling , and T. Cremer , Lab Invest. 74, 108 (1996). 8569172

[3] S. A. Grossman and S. G. Ellsworth , J. Clin. Oncol. 34, e13522 (2016). 10.1200/JCO.2016.34.15_suppl.e13522

[4] TCGA Research Network, Nature 455, 1061 (2008). 10.1038/nature07385 18772890 PMC2671642

[5] TCGA Research Network, N. Engl. J. Med. 372, 2481 (2015). 10.1056/NEJMoa1402121 26061751 PMC4530011

[6] C. H. Lee , B. O. Alpert , P. Sankaranarayanan , and O. Alter , PLoS One 7, e30098 (2012). 10.1371/journal.pone.0030098 22291905 PMC3264559

[7] K. A. Aiello and O. Alter , PLoS One 11, e0164546 (2016). 10.1371/journal.pone.0164546 27798635 PMC5087864

[8] Q. T. Ostrom , H. Gittleman , P. Liao , T. Vecchione-Koval , Y. Wolinsky , C. Kruchko , and J. S. Barnholtz-Sloan , Neuro-Oncology 19, v1 (2017). 10.1093/neuonc/nox158 29117289 PMC5693142

[9] K. A. Aiello , S. P. Ponnapalli , and O. Alter , in 2018 AACR Annual Meeting, 14–18 April (2018).

[10] G. Klambauer , K. Schwarzbauer , A. Mayr , D. A. Clevert , A. Mitterecker , U. Bodenhofer , and S. Hochreiter , Nucleic Acids Res. 40, e69 (2012). 10.1093/nar/gks003 22302147 PMC3351174

[11] D. Karolchik , G. P. Barber , J. Casper , H. Clawson , M. S. Cline , M. Diekhans , T. R. Dreszer , P. A. Fujita , L. Guruvadoo , M. Haeussler , R. A. Harte , S. Heitner , A. S. Hinrichs , K. Learned , B. T. Lee , C. H. Li , B. J. Raney , B. Rhead , K. R. Rosenbloom , C. A. Sloan , M. L. Speir , A. S. Zweig , D. Haussler , R. M. Kuhn , and W. J. Kent , Nucleic Acids Res. 42, D764 (2014). 10.1093/nar/gkt1168 24270787 PMC3964947

[12] R. R. Haraksingh , A. Abyzov , and A. E. Urban , BMC Genomics 18, 321 (2017). 10.1186/s12864-017-3658-x 28438122 PMC5402652

[13] R. Shen and V. E. Seshan , Nucleic Acids Res. 44, e131 (2016). 10.1093/nar/gkw520 27270079 PMC5027494

[14] R. J. Roberts , M. O. Carneiro , and M. C. Schatz , Genome Biol. 14, 405 (2013). 10.1186/gb-2013-14-6-405 23822731 PMC3953343

[15] D. Pinto , K. Darvishi , X. Shi , D. Rajan , D. Rigler , T. Fitzgerald , A. C. Lionel , B. Thiruvahindrapuram , J. R. Macdonald , R. Mills , A. Prasad , K. Noonan , S. Gribble , E. Prigmore , P. K. Donahoe , R. S. Smith , J. H. Park , M. E. Hurles , N. P. Carter , C. Lee , S. W. Scherer , and L. Feuk , Nat. Biotechnol. 29, 512 (2011). 10.1038/nbt.1852 21552272 PMC3270583

[16] C. F. Van Loan , SIAM J. Numer. Anal. 13, 76 (1976). 10.1137/0713009

[17] C. C. Paige and M. A. Saunders , SIAM J. Numer. Anal. 18, 398 (1981). 10.1137/0718026

[18] S. Friedland , SIAM J. Matrix Anal. Appl. 27, 434 (2005). 10.1137/S0895479804439791

[19] R. A. Horn and C. R. Johnson , *Matrix Analysis*, 2nd ed ( Cambridge University Press, Cambridge, UK, 2012).

[20] G. H. Golub and C. F. Van Loan , *Matrix Computations*, 4th ed ( Johns Hopkins University Press, Baltimore, MD, 2012).

[21] H. Goldstein , *Classical Mechanics*, 2nd ed ( Addison-Wesley, Reading, MA, 1980).

[22] O. Alter , P. O. Brown , and D. Botstein , Proc. Natl. Acad. Sci. USA 100, 3351 (2003). 10.1073/pnas.0530258100 12631705 PMC152296

[23] O. Alter , G. H. Golub , P. O. Brown , and D. Botstein , in Miami Nature Biotechnology Winter Symposium on Cell Cycle, Chromosomes and Cancer, 31 January–4 February (2004).

[24] S. P. Ponnapalli , G. H. Golub , and O. Alter , in Stanford University and Yahoo! Research Workshop on Algorithms for Modern Massive Datasets, 21–24 June (2006).

[25] S. P. Ponnapalli , M. A. Saunders , C. F. Van Loan , and O. Alter , PLoS One 6, e28072 (2011). 10.1371/journal.pone.0028072 22216090 PMC3245232

[26] P. Sankaranarayanan , T. E. Schomay , K. A. Aiello , and O. Alter , PLoS One 10, e0121396 (2015). 10.1371/journal.pone.0121396 25875127 PMC4398562

[27] K. A. Aiello , C. A. Maughan , T. E. Schomay , S. P. Ponnapalli , H. A. Hanson , and, O. Alter , in 2018 AACR Annual Meeting, 14–18 April (2018).

[28] L. N. Trefethen and D. Bau III , *Numerical Linear Algebra* ( SIAM, Philadelphia, PA,1997).

[29] A. Edelman , T. A. Arias , and S. T. Smith , SIAM J. Matrix Anal. Appl. 20, 303 (1998). 10.1137/S0895479895290954

[30] L. M. Ewerbring and F. T. Luk , J. Comput. Appl. Math. 27, 37 (1989). 10.1016/0377-0427(89)90360-9

[31] A. B. Olshen , E. S. Venkatraman , R. Lucito , and M. Wigler , Biostatistics 5, 557 (2004). 10.1093/biostatistics/kxh008 15475419

[32] L. A. Lettice , T. Horikoshi , S. J. Heaney , M. J. van Baren , H. C. van der Linde , G. J. Breedveld , M. Joosse , N. Akarsu , B. A. Oostra , N. Endo , M. Shibata , M. Suzuki , E. Takahashi , T. Shinka , Y. Nakahori , D. Ayusawa , K. Nakabayashi , S. W. Scherer , P. Heutink , R. E. Hill , and S. Noji , Proc. Natl. Acad. Sci. USA 99, 7548 (2002). 10.1073/pnas.112212199 12032320 PMC124279

[33] B. W. Purow , R. M. Haque , M. W. Noel , Q. Su , M. J. Burdick , J. Lee , T. Sundaresan , S. Pastorino , J. K. Park , I. Mikolaenko , D. Maric , C. G. Eberhart , and H. A. Fine , Cancer Res. 65, 2353 (2005). 10.1158/0008-5472.CAN-04-1890 15781650

[34] W. Sun , D. A. Gaykalova , M. F. Ochs , E. Mambo , D. Arnaoutakis , Y. Liu , M. Loyo , N. Agrawal , J. Howard , R. Li , S. Ahn , E. Fertig , D. Sidransky , J. Houghton , K. Buddavarapu , T. Sanford , A. Choudhary , W. Darden , A. Adai , G. Latham , J. Bishop , R. Sharma , W. H. Westra , P. Hennessey , C. H. Chung , and J. A. Califano , Cancer Res. 74, 1091 (2014). 10.1158/0008-5472.CAN-13-1259 24351288 PMC3944644

[35] G. Laureys , F. Speleman , R. Versteeg , P. van der Drift , A. Chan , J. Leroy , U. Francke , G. Opdenakker , and N. Van Roy , Oncogene 10, 1087 (1995). 7700633

[36] M. O'Bleness , V. B. Searles , C. M. Dickens , D. Astling , D. Albracht , A. C. Mak , Y. Y. Lai , C. Lin , C. Chu , T. Graves , P. Y. Kwok , R. K. Wilson , and J. M. Sikela , BMC Genomics 15, 387 (2014). 10.1186/1471-2164-15-387 24885025 PMC4053653

[37] S. Weijzen , P. Rizzo , M. Braid , R. Vaishnav , S. M. Jonkheer , A. Zlobin , B. A. Osborne , S. Gottipati , J. C. Aster , W. C. Hahn , M. Rudolf , K. Siziopikou , W. M. Kast , and L. Miele , Nat. Med. 8, 979 (2002). 10.1038/nm754 12185362

[38] W. C. Hahn , C. M. Counter , A. S. Lundberg , R. L. Beijersbergen , M. W. Brooks , and R. A. Weinberg , Nature 400, 464 (1999). 10.1038/22780 10440377

[39] H. Kiaris , K. Politi , L. M. Grimm , M. Szabolcs , P. Fisher , A. Efstratiadis , and S. Artavanis-Tsakonas , Am. J. Pathol. 165, 695 (2004). 10.1016/S0002-9440(10)63333-0 15277242 PMC1618582

[40] M. E. Carlson , M. Hsu , and I. M. Conboy , Nature 454, 528 (2008). 10.1038/nature07034 18552838 PMC7761661

[41] T. Waldman , C. Lengauer , K. W. Kinzler , and B. Vogelstein , Nature 381, 713 (1996). 10.1038/381713a0 8649519

[42] J. Irianto , Y. Xia , C. R. Pfeifer , A. Athirasala , J. Ji , C. Alvey , M. Tewari , R. R. Bennett , S. M. Harding , A. J. Liu , R. A. Greenberg , and D. E. Discher , Curr. Biol. 27, 210 (2017). 10.1016/j.cub.2016.11.049 27989676 PMC5262636

[43] J. H. Kong , L. Yang , E. Dessaud , K. Chuang , D. M. Moore , R. Rohatgi , J. Briscoe , and B. G. Novitch , Dev. Cell 33, 373 (2015). 10.1016/j.devcel.2015.03.005 25936505 PMC4449290

[44] R. Rohatgi and M. P. Scott , Nat. Cell Biol. 9, 1005 (2007). 10.1038/ncb435 17762891

[45] E. Y. Lee , H. Ji , Z. Ouyang , B. Zhou , W. Ma , S. A. Vokes , A. P. McMahon , W. H. Wong , and M. P. Scott , Proc. Natl. Acad. Sci. USA 107, 9736 (2010). 10.1073/pnas.1004602107 20460306 PMC2906878

[46] I. T. Fiddes , G. A. Lodewijk , M. M. Mooring , C. M. Bosworth , A. D. Ewing , G. L. Mantalas , A. M. Novak , A. van den Bout , A. Bishara , J. L. Rosenkrantz , R. Lorig-Roach , A. R. Field , M. Haeussler , L. Russo , A. Bhaduri , T. J. Nowakowski , A. A. Pollen , M. L. Dougherty , X. Nuttle , M. C. Addor , S. Zwolinski , S. Katzman , A. Kreigstein , E. E. Eichler , S. R. Salama , F. M. J. Jacobs , and D. Haussler , Cell 173, 1356 (2018). 10.1016/j.cell.2018.03.051 29856954 PMC5986104

[47] M. C. Popesco , E. J. Maclaren , J. Hopkins , L. Dumas , M. Cox , L. Meltesen , L. McGavran , G. J. Wyckoff , and J. M. Sikela , Science 313, 1304 (2006). 10.1126/science.1127980 16946073

[48] U. Fischer , P. Meltzer , and E. Meese , Hum. Genet. 98, 625 (1996). 10.1007/s004390050271 8882887

[49] M. G. Netsky , B. August , and W. Fowler , J. Neurosurg. 7, 261 (1950). 10.3171/jns.1950.7.3.0261 15415784

[50] P. Bady , D. Sciuscio , A. C. Diserens , J. Bloch , M. J. van den Bent , C. Marosi , P. Y. Dietrich , M. Weller , L. Mariani , F. L. Heppner , D. R. Mcdonald , D. Lacombe , R. Stupp , M. Delorenzi , and M. E. Hegi , Acta Neuropathol. 124, 547 (2012). 10.1007/s00401-012-1016-2 22810491 PMC3444709

[51] C. W. Brennan , R. G. Verhaak , A. McKenna , B. Campos , H. Noushmehr , S. R. Salama , S. Zheng , D. Chakravarty , J. Z. Sanborn , S. H. Berman , R. Beroukhim , B. Bernard , C. J. Wu , G. Genovese , I. Shmulevich , J. S. Barnholtz-Sloan , L. Zou , R. Vegesna , S. A. Shukla , G. Ciriello , W. K. Yung , W. Zhang , C. Sougnez , T. Mikkelsen , K. Aldape , D. D. Bigner , E. G. Van Meir , M. Prados , A. Sloan , K. L. Black , J. Eschbacher , G. Finocchiaro , W. Friedman , D. W. Andrews , A. Guha , M. Iacocca , B. P. O'Neill , G. Foltz , J. Myers , D. J. Weisenberger , R. Penny , R. Kucherlapati , C. M. Perou , D. N. Hayes , R. Gibbs , M. Marra , G. B. Mills , E. Lander , P. Spellman , R. Wilson , C. Sander , J. Weinstein , M. Meyerson , S. Gabriel , P. W. Laird , D. Haussler , G. Getz , L. Chin , and TCGA Research Network, Cell 155, 462 (2013). 10.1016/j.cell.2013.09.034 24120142 PMC3910500

[52] P. Howland and H. Park , IEEE Trans. Pattern Anal. Mach. Intell. 26, 995 (2004). 10.1109/TPAMI.2004.46 15641730

[53] J. A. Berger , S. Hautaniemi , S. K. Mitra , and J. Astola , IEEE/ACM Trans. Comput. Biol. Bioinf. 3, 2 (2006). 10.1109/TCBB.2006.10 17048389

[54] W. De Clercq , A. Vergult , B. Vanrumste , W. Van Paesschen , and S. Van Huffel , IEEE Trans. Biomed. Eng. 53, 2583 (2006). 10.1109/TBME.2006.879459 17153216

[55] A. E. Teschendorff , M. Journée , P. A. Absil , R. Sepulchre , and C. Caldas , PLoS Comput. Biol. 3, e161 (2007). 10.1371/journal.pcbi.0030161 17708679 PMC1950343

[56] A. W. Schreiber , N. J. Shirley , R. A. Burton , and G. B. Fincher , BMC Bioinf. 9, 335 (2008). 10.1186/1471-2105-9-335 PMC2562393 18687147

[57] I. Rustandi , M. A. Just , and T. M. Mitchell , in *Proceedings of the MICCAI Workshop on Statistical Modeling and Detection Issues in Intra-and Inter-Subject Functional MRI Data Analysis*, 20–24 September (2009).

[58] X. Xiao , N. Dawson , L. Macintyre , B. J. Morris , J. A. Pratt , D. G. Watson , and D. J. Higham , BMC Syst. Biol. 5, 72 (2011). 10.1186/1752-0509-5-72 21575198 PMC3239845

[59] O. A. Tomescu , D. Mattanovich , and G. G. Thallinger , BMC Syst. Biol. 8, S4 (2014). 10.1186/1752-0509-8-S2-S4 PMC4101701 25033389

[60] X. Xiao , A. Moreno-Moral , M. Rotival , L. Bottolo , and E. Petretto , PLoS Genet. 10, e1004006 (2014). 10.1371/journal.pgen.1004006 24391511 PMC3879165

[61] T. Adali , Y. Levin-Schwartz , and V. D. Calhoun , Proc. IEEE 103, 1478 (2015). 10.1109/JPROC.2015.2461624 PMC4624202 26525830

[62] X. Chen , Z. J. Wang , and M. McKeown , IEEE Signal Process. Mag. 33, 86 (2016). 10.1109/MSP.2016.2521870

[63] Y. Wang , W. Zhao , and X. Zhou , Sci. Rep. 6, 34335 (2016). 10.1038/srep34335 27703186 PMC5050522

[64] Z. Chitforoushzadeh , Z. Ye , Z. Sheng , S. LaRue , R. C. Fry , D. A. Lauffenburger , and K. A. Janes , Sci. Signal. 9, ra59 (2016). 10.1126/scisignal.aad3373 27273097 PMC4914393

[65] R. Redon , S. Ishikawa , K. R. Fitch , L. Feuk , G. H. Perry , T. D. Andrews , H. Fiegler , M. H. Shapero , A. R. Carson , W. Chen , E. K. Cho , S. Dallaire , J. L. Freeman , J. R. González , M. Gratacòs , J. Huang , D. Kalaitzopoulos , D. Komura , J. R. MacDonald , C. R. Marshall , R. Mei , L. Montgomery , K. Nishimura , K. Okamura , F. Shen , M. J. Somerville , J. Tchinda , A. Valsesia , C. Woodwark , F. Yang , J. Zhang , T. Zerjal , J. Zhang , L. Armengol , D. F. Conrad , X. Estivill , C. Tyler-Smith , N. P. Carter , H. Aburatani , C. Lee , K. W. Jones , S. W. Scherer , and M. E. Hurles , Nature 444, 444 (2006). 10.1038/nature05329 17122850 PMC2669898

[66] J. R. Lupski , Nat. Genet. 39, S43 (2007). 10.1038/ng2084 17597781

[67] S. J. Diskin , C. Hou , J. T. Glessner , E. F. Attiyeh , M. Laudenslager , K. Bosse , K. Cole , Y. P. Mossé , A. Wood , J. E. Lynch , K. Pecor , M. Diamond , C. Winter , K. Wang , C. Kim , E. A. Geiger , P. W. McGrady , A. I. Blakemore , W. B. London , T. H. Shaikh , J. Bradfield , S. F. Grant , H. Li , M. Devoto , E. R. Rappaport , H. Hakonarson , and J. M. Maris , Nature 459, 987 (2009). 10.1038/nature08035 19536264 PMC2755253

[68] E. Vanneste , T. Voet , C. Le Caignec , M. Ampe , P. Konings , C. Melotte , S. Debrock , M. Amyere , M. Vikkula , F. Schuit , J. P. Fryns , G. Verbeke , T. D'Hooghe , Y. Moreau , and J. R. Vermeesch , Nat. Med. 15, 577 (2009). 10.1038/nm.1924 19396175

[69] U. Fischer , A. Keller , M. Voss , C. Backes , C. Welter , and E. Meese , PLoS One 7, e37422 (2012). 10.1371/journal.pone.0037422 22606362 PMC3351388

